# Characteristics of mental health implications and plasma metabolomics in patients recently recovered from COVID-19

**DOI:** 10.1038/s41398-021-01426-3

**Published:** 2021-05-21

**Authors:** Lian Yang, Mei Zhou, Lingli Li, Ping Luo, Wenliang Fan, Juanjuan Xu, Qing Chen, Feng Pan, Ping Lei, Chuansheng Zheng, Yang Jin

**Affiliations:** 1 Department of Radiology, Union Hospital, Tongji Medical College, Huazhong University of Science and Technology, 430022 Wuhan, China; 2 Hubei Province Key Laboratory of Molecular Imaging, 430022 Wuhan, China; 3 Department of Respiratory and Critical Care Medicine, NHC Key Laboratory of Pulmonary Diseases, Union Hospital, Tongji Medical College, Huazhong University of Science and Technology, 430022 Wuhan, Hubei China; 4 Department of Translational Medicine Center, Union Hospital, Tongji Medical College, Huazhong University of Science and Technology, 430022 Wuhan, Hubei China; 5 Health Checkup Department, Union Hospital, Tongji Medical College, Huazhong University of Science and Technology, 430022 Wuhan, Hubei China; 6 Department of Immunology, School of Basic Medicine, Tongji Medical College, Huazhong University of Science and Technology, 430022 Wuhan, Hubei China

**Keywords:** Human behaviour, Diagnostic markers, Molecular neuroscience

## Abstract

This study aimed to explore the associations between cerebral white matter (WM) alterations, mental health status, and metabolism in recovered COVID-19 patients. We included 28 recovered COVID-19 patients and 27 healthy controls between April 2020 and June 2020. Demographic data, the mental health scores, diffusion-tensor imaging (DTI) data, and plasma metabolomics were collected and compared between the two groups. Tract-based spatial statistics and graph theory approaches were used for DTI data analysis. Untargeted metabolomics analysis of the plasma was performed. Correlation analyses were performed between these characteristics. Recovered COVID-19 patients showed decreased fractional anisotropy, increased mean diffusivity and radial diffusivity values in widespread brain regions, and significantly lower global efficiency, longer shortest path length, and less nodal local efficiency in superior occipital gyrus (all, *P* < 0.05, Bonferroni corrected). Our results also demonstrated significantly different plasma metabolic profiling in recovered COVID-19 patients even at 3 months after their hospital discharge, which was mainly related to purine pathways, amino acids, lipids, and amine metabolism. Certain regions with cerebral WM alterations in the recovered patients showed significant correlations with different metabolites and the mental health scores. We observed multiple alterations in both WM integrity and plasma metabolomics that may explain the deteriorated mental health of recovered COVID-19 patients. These findings may provide potential biomarkers for the mental health evaluation for the recovered COVID-19 patients and potential targets for novel therapeutics.

## Introduction

As of November 24, 2020, coronavirus disease 2019 (COVID-19) pandemic, caused by severe acute respiratory syndrome coronavirus 2 (SARS-CoV-2), has resulted in more than 57 million confirmed cases worldwide, with a mortality rate as high as 2.4%, and the number of recovered cases at more than 56 million worldwide^1^. Although the main clinical manifestation is respiratory symptoms, reports of neurological presentations are increasing^2–4^. Regarding the evidence from other corona-viruses infected humans, the minimum prevalence of central nervous system (CNS) complications ranged from 0.04% for the severe acute respiratory syndrome (SARS) to 0.20% for the Middle East respiratory syndrome (MERS)^2^. We, therefore, speculate the scale of the current pandemic to build up a large number of cases with CNS complications even if the proportion is small, and their associated health burden and social and economic costs might be large. Previous studies^2–6^ have revealed different neurological manifestations and CNS complications in hospitalized patients with COVID-19 or during the treatment period.

As the pandemic of COVID-19 is ongoing, there has been a growing recognition of the mental health implications of the disease^7,8^. Coronaviruses have been shown to be potentially neurotropic, neurovirulent, and neuroinvasive, including SARS coronavirus and MERS coronavirus^9^. Neurological symptoms of patients with COVID-19, such as seizures and anosmia during infection might reflect CNS involvement^4^. A previous meta-analysis of the mental health implications in patients admitted to hospital with SARS and MERS showed that the point prevalence in the post-illness stage was 32.2% for post-traumatic stress disorder, 14.9% for depression, and 14.8% for anxiety^8^. Recently, a study reported the neuropsychiatric sequelae of acutely ill COVID-19 inpatients in isolation facilities, include the clinically significant symptoms of posttraumatic stress disorder, depression, anxiety, and stress^10^. The analysis for the mental health implications in recovered COVID-19 patients without previous underlying diseases after hospital discharge is therefore paramount and urgent.

On the other hand, increasing studies have revealed that the metabolic profiling of blood is sensitive to catch the biological states related to mental disorders, and even able to identify potential blood-based biomarkers of psychiatric diseases^11^. Recent studies reported that COVID-19 patients present obvious blood metabolic dysregulation, and metabolic alterations were closely correlated with disease severity^12,13^. Additionally, discharged COVID-19 patients were also found with disordered plasma metabolism, which aligns with the progress and severity of this disease^14^. However, the metabolic alterations that may underly the mental health implications in recovered COVID-19 patients have remained poorly understudied.

We, therefore, hypothesize the characteristics of cerebral white matter (WM) and blood metabolome may provide novel insight into the mental health implications and mechanism in recovered COVID-19 patients. We evaluated the cerebral WM changes by the topologic alterations in the WM network and anisotropy in multiple WM tracts in recovered COVID-19 patients without previous underlying diseases at 3 months after their hospital discharge. Additionally, plasma metabolomics was utilized to investigate the potential underlying molecule mechanism from the metabolite level. The associations between cerebral WM, mental health status, and metabolites were further explored to enhance the understanding of the mental health implications.

## Methods

### Study design and participants

The Ethics Committee of Union Hospital, Tongji Medical College, Huazhong University of Science and Technology (No. 2020-0036) has approved this prospective cohort (NCT04283396, ChiCTR2000031356) study. All of the participants included in this study were informed about the purpose of the research before giving written consent in accordance with Chinese legislation, and with the Declaration of Helsinki.

The flow chart of participants recruitment was depicted in the appendix Fig. S1. For details about the inclusion and exclusion criteria, see the appendix. From April 2020 to June 2020, finally, a total of 28 recovered COVID-19 patients without previous underlying diseases at 3 months after their hospital discharge and 27 healthy controls (HC) admitted to this single-center were enrolled in the study. The discharge criteria for these conformed to the published standard protocols from the National Health Commission of the People’s Republic of China^15^. The two participant groups had a similar experience with the outbreak of COVID-19 in Wuhan and homogenous demographic characteristics.

The mental health statuses of the recovered COVID-19 patients and HC were assessed by an experienced psychiatrist using the 9-Item Patient Health Questionnaire (PHQ-9), the Generalized Anxiety Disorder Screener GAD-7, the Posttraumatic Stress Disorder Self-Rating Scale (PTSD-SS), the PTSD checklist-civilian version (PCL-C), the Hamilton Anxiety Rating Scale (HAMA), and the 17-item Hamilton Depression Rating Scale (HAMD).

All participants underwent blood tests, including complete blood count, renal and liver function, coagulation profile, creatine kinase. Simultaneously, we collected their peripheral blood for subsequent untargeted metabolomic determination.

### Image acquisition and data preprocessing

Details are provided in the appendix. All of the participants were imaged these MRI sequences using a Siemens 3 T scanner (Magnetom Skyra; Siemens, Erlangen, Germany): T1-weighted MPRAGE sequence (1 mm isotropic); Diffusion-tensor MRI sequence (*b* values = 0 and 1000 s/mm^2^ along 64 directions, voxel size = 2 mm isotropic); and T2-weighted FLAIR sequence. Diffusion-tensor imaging (DTI) preprocessing and WM network construction were performed by using FSL^16^ and PANDA software (http://www.nitrc.org/projects/panda/). Data preprocessing of various DTI-derived indies (FA: fractional anisotropy; MD: mean diffusivity; AD: axial diffusivity and RD: radial diffusivity) was performed using tract-based spatial statistics (TBSS) and FSL. The diffusion-weighted images were corrected for eddy current distortions and head motion with the FDT toolkit available in FSL. First, the FA images were created by fitting a tensor model to the raw diffusion data using FDT, followed by the brain extraction process using BET. Second, the FA data from all subjects were aligned to the MNI space. Third, a mean FA image was generated to produce a mean FA skeleton, which represented the centers of all tracts common to the group. Finally, each subject’s aligned FA data were projected onto the skeleton, and the resulting data were fed into voxelwise cross-subject statistical analyses. In the same way, the MD, AD, and RD images were aligned to the MNI space and then were projected onto the mean FA skeleton by using the protocol for non-FA images in TBSS. More detailed preprocessing can be seen in the appendix and our previous study^17^.

### Network construction and analysis

Deterministic DTI tractography was performed with the Diffusion Toolkit and Trackvis (https://www.nitrc.org/projects/trackvis) by using the Fiber Assignment by Continuous Tracking (or FACT) algorithm to depict the whole WM network as a connectivity matrix of 90 regions (derived from Automated Anatomic Labeling atlas). For details about how to construct the WM network, see the appendix. All network analyses were performed using the Gretna software (http://www.nitrc.org/projects/gretna/) and viewed on the BrainNet Viewer software (http://www.nitrc.org/projects/bnv/). To characterize the topologic organization of the WM network, the following graph metrics were assessed: global efficiency (Eglob), local efficiency (Eloc), shortest path length (Lp), clustering coefficient (Cp), and small-world parameters (*γ*, *λ*, and *σ*). For nodal parameters, we calculated four nodal centrality metrics: betweenness centrality (Bi), degree centrality (Di), nodal local efficiency, and nodal efficiency.

### Determination of plasma metabolites

All plasma samples were collected after overnight fasting and stored at −80 °C. Three volumes of methanol (−20 °C) were added to 100 µL thawed plasma (4 °C), and the mixture was vortexed for 1 min and equilibrating for 20 min. Then the mixture was centrifuged at 10,000 × *g* (4 °C, 10 min), and the supernatant was dried under a stream of nitrogen. Finally, the dried sample was dissolved in ultrapure water before further LC–MS analysis.

The acquisition was performed with an ultra-high performance liquid chromatography (Ultimate 3000, hermo Scientific, USA) system coupled to a Q Exactive high-resolution mass spectrometry (HRM) system (Thermo Scientific, USA). Waters ACQUITY UPLC HSS T3 columns (1.8 μm, 100 mm × 2.1 mm ID) were used in both positive and negative ion mode acquisitions. Detailed parameters of LC separation and MS detection are described in the appendix.

The raw data were converted by AnalysisBase File Converter software, and processed with MS-DIAL (http://prime.psc.riken.jp/compms/msdial/main.html) version 4.12 according to the user guide. All metabolites were identified by online public available databases (MSBank (http://www.massbank.jp/) and HMDB (https://hmdb.ca/)).

### Statistical analysis

All the statistical analyses of demographic information were performed using IBM SPSS Statistics Software (version 24; IBM, New York, USA). Demographic factors and clinical variables including age, sex, body mass index (BMI), years of education, PHQ-9, GAD-7, HAMA, HAMD, PCL-C, and PTSD-SS scores were compared between the two groups based on the variable types and their distributions.

To assess the statistical significance of between-group comparisons of the network metrics and the measures of various DTI-derived indices (FA, AD, RD, and MD), permutation-based non-parametric inferences were performed between the two groups (5000 random permutations) with the sex, age, BMI, and years of education as covariates. For the multiple comparison corrections, the threshold-free cluster enhancement (TFCE) with FWE (*P* < 0.05) was employed. In addition, the correlations between network metrics, the various DTI-derived indices (FA, AD, RD, and MD), and the mental health scores were performed by Spearman correlation analysis.

Metabolome statistical analyses were carried out by Metaboanalyst (https://www.metaboanalyst.ca/). Significance was analyzed by using *t*-test and Fold changes. Metabolites with an FDR-adjusted *P* value < 0.01, FC > 2 or FC < 0.5, VIP > 1 are considered as statistically significant. Pearson correlation analysis between metabolites and network metrics, the various DTI-derived indices (FA, AD, RD, and MD) were carried out by open-source R pheatmap package, and its network was displayed by using ggplot2 (open-source R igraph package).

## Results

### Demographic characteristic and clinical features of recovered COVID-19 patients

There were no differences in age, sex, BMI, and years of education between recovered COVID-19 patients and the HC groups (all, *P* > 0.05). For the mental health scores, no inter-group differences were found in PHQ-9 (*P* > 0.05). However, recovered COVID-19 patients had significantly higher scores in the PCL-C, PTSD-SS, GAD-7, HAMA, and HAMD (all, *P* < 0.05) (Table 1). The appendix Table S1 showed the laboratory findings in recovered COVID-19 patients and HC. Compared to HC, white blood cells and neutrophils were significantly decreased, while albumin was significantly increased (all, *P* < 0.05) (the appendix Table S1).

**Table 1:** Summary of the demographic and clinical data.

Demographic	Recovered COVID-19 patients (*n* = 28)	Healthy controls (*n* = 27)	*P*-value
Age, years	40 ± 7.9	37.7 ± 9.0	0.32^a^
*Sex*			
Female	16 (57%)	14 (52%)	0.69^b^
Male	12 (43%)	13 (48%)	
BMI	24.3 ± 2.6	23.2 ± 3.2	0.14^a^
Education, years	15 (12–16)	15 (9–17)	0.48^c^
*Mental health scores*			
PHQ-9	2.5 (0–5.25)	2 (0.5–4)	0.49^c^
GAD-7	1.5 (0–4)	0 (0–1.5)	<0.05^c^
HAMD	5 (2–9.25)	2 (1–4.5)	<0.01^c^
HAMA	2.5 (1–5.25)	1 (0–3)	<0.05^c^
PCL-C	23 (19–27.5)	18 (17–18.5)	<0.01^c^
PTSD-SS	29.5 (26.25–38)	24 (24–25)	<0.01^c^
*Nervous system symptoms*			
Dizziness	3 (11%)	0	NA
Headache	2 (7%)	0	NA
Nerve pain	2 (7%)	0	NA
*Impairment*			
Taste	2 (7%)	0	NA
Smell	2 (7%)	0	NA
Vision	4 (14%)	0	NA

Data are mean ± SD, *n* (%) or median (IQR), unless otherwise specified.

*NA* not applicable, *BMI* body mass index, *PHQ-9* the 9-Item Patient Health Questionnaire, *GAD-7* the Generalized Anxiety Disorder Screener GAD-7, *PTSD-SS* the Posttraumatic Stress Disorder Self-Rating Scale, *PCL-C* the PTSD checklist-civilian version, *HAMA* the Hamilton Anxiety Rating Scale, *HAMD* the 17-item Hamilton Depression Rating Scale.

^a^Independent sample *t* test.

^b^Chi-square test.

^c^Mann-Whitney *U* test.

### DTI-derived indices and DTI network metrics

**Fig. 1:**
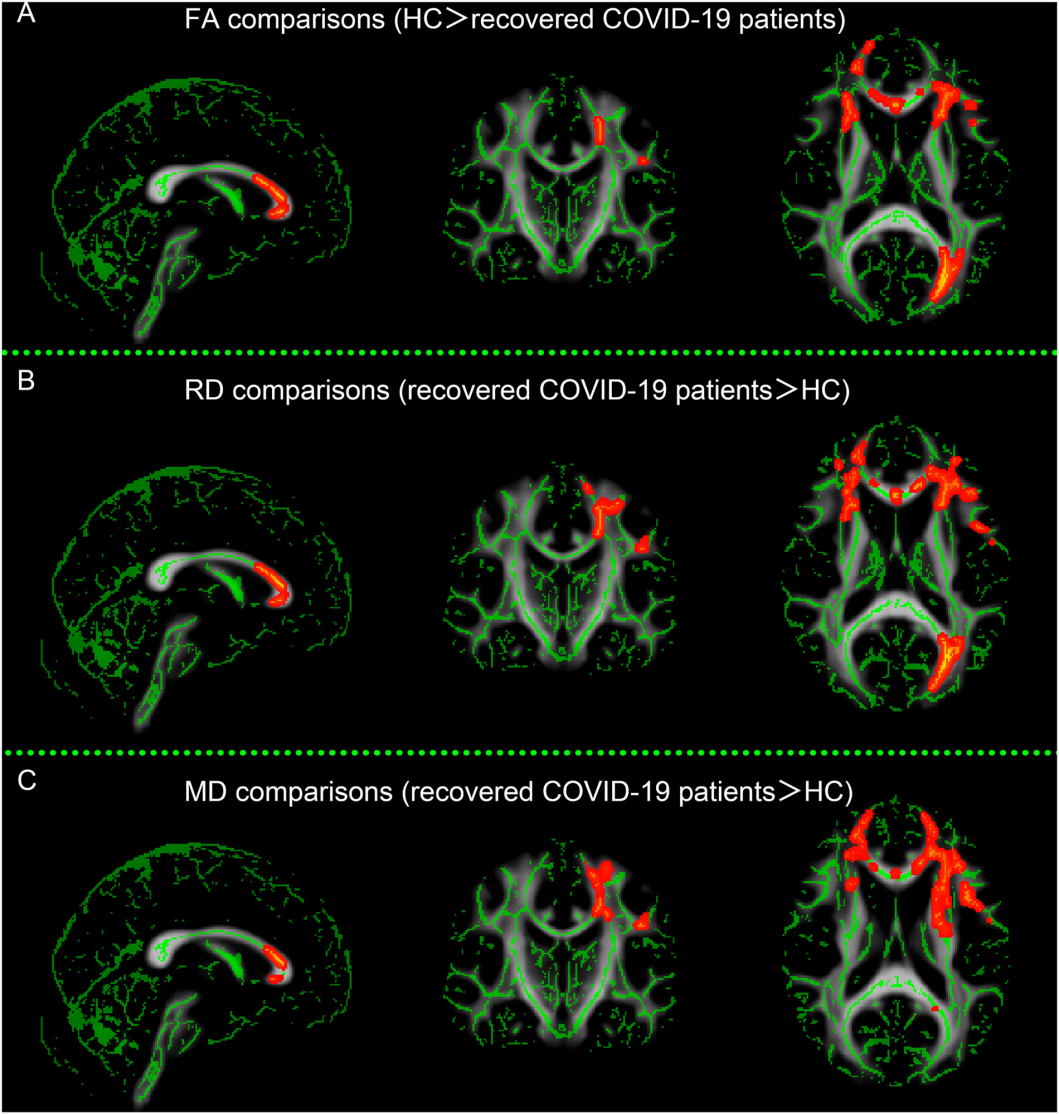
Differences in the white matter microstructures between recovered COVID-19 patients and healthy controls (HC). Compared with HC, recovered COVID-19 patients showed significantly decreased fractional anisotropy (FA) (**A**), increased radial diffusivity (RD) (**B**), and increased mean diffusivity (MD) (**C**) in multiple brain regions (*P* < 0.05, TFCE corrected), whereas no difference in axial diffusivity (AD) was observed.

Compared to the HC, the recovered COVID-19 patients showed decreased FA, increased MD and RD values in multiple WM tracts, including the corpus callosum, corona radiate, internal capsule, external capsule, superior longitudinal fasciculus, left posterior thalamic radiation, left cingulum, and left superior fronto-occipital fasciculus, while there were no significant differences in the AD values (Fig. 1).

DTI network metrics included global network properties and nodal parameters. The results of the analysis of global topologic network properties were reported. Compared to the HC, recovered COVID-19 patients exhibited significantly lower Eglob (*P* < 0.05, Bonferroni corrected) and longer Lp (*P* < 0.05, Bonferroni corrected). Compared with HC, the recovered COVID-19 patients exhibited less nodal local efficiency in the superior occipital gyrus (*P* < 0.05, Bonferroni corrected).

### Correlations between DTI network metrics, the various DTI-derived indices, and the mental health scores

**Fig. 2:**
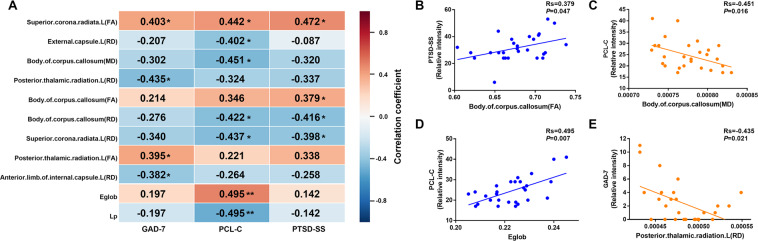
Correlations between DTI network metrics, the various DTI-derived indices, and the mental health scores. **A** Correlations between DTI network metrics, the various DTI-derived indices, and the mental health scores in recovered COVID-19 patients. Each cell contains the correlation coefficient corresponding to the color scale. **B** Scatter plot of the body of corpus callosum (FA) and PTSD-SS score. **C** Scatter plot of the body of corpus callosum (MD) and PCL-C score. **D** Scatter plot of Eglob and PCL-C score. **E** Scatter plot of left posterior thalamic radiation (RD) and GAD-7 score. Eglob global efficiency, Lp shortest path length, GAD-7 the Generalized Anxiety Disorder Screener GAD-7, PCL-C the PTSD checklist-civilian version, PTSD-SS the Posttraumatic Stress Disorder Self-Rating Scale, Rs Spearman’s correlation coefficient. *P < 0.05; **P < 0.01.

A correlation analysis showed that the FA values in the left superior corona radiate, the body of corpus callosum, and left posterior thalamic radiation showed significant positive correlations with the PCL-C, PTSD-SS, and GAD-7 scores (Fig. 2). RD values in the left superior corona radiate, body of corpus callosum, left external capsule, left anterior limb of the internal capsule and posterior thalamic radiation showed significant negative correlations with the PCL-C, PTSD-SS, and GAD-7 scores (Fig. 2). MD values in the body of the corpus callosum showed significant negative correlations with the PCL-C (Fig. 2). Eglob showed significant positive correlations with the PCL-C (*P* = 0.007, *r* = 0.495), while Lp showed significant negative correlations with the PCL-C (*P* = 0.007, *r* = −0.495) (Fig. 2).

### Recovered COVID-19 patients presented plasma metabolic profiles that were different from HC

**Fig. 3:**
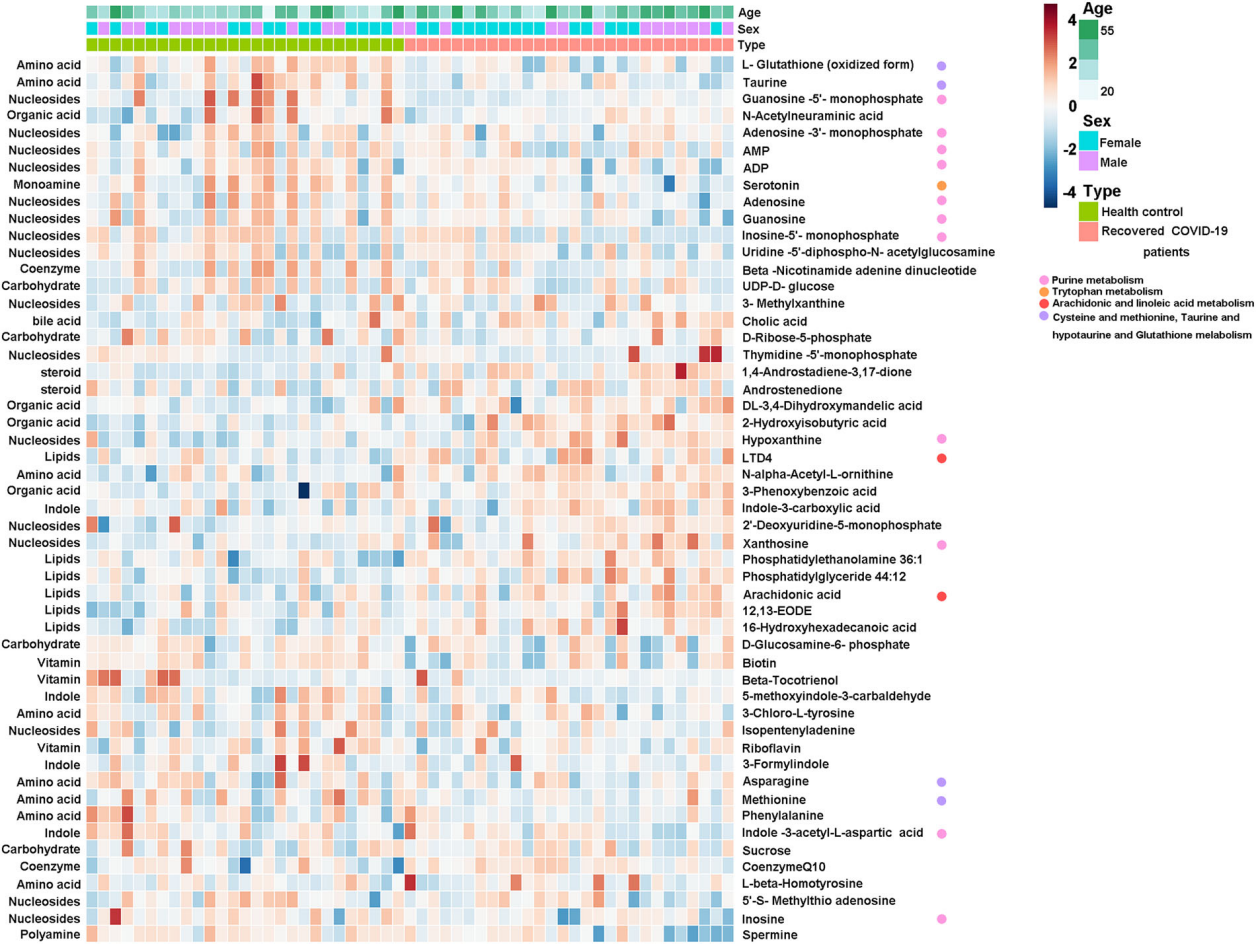
Differential plasma metabolites revealed in recovered COVID-19 patients when compared with healthy controls. Heat map of 52 differential metabolites from 12 major classes in recovered COVID-19 patients, metabolites levels were displayed by the shades of the color (red and blue present lower level, and higher level, respectively).

**Fig. 4:**
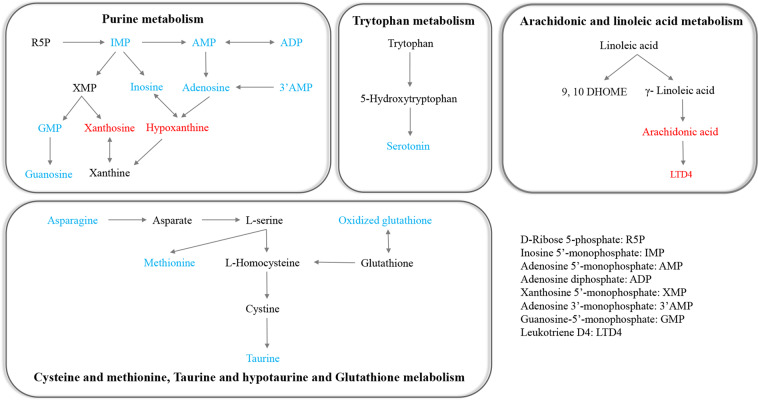
Main disordered pathways of differential metabolites in recovered COVID-19 patients when compared to healthy controls 3 months after discharge. The color of red and blue for metabolites illustrated that the metabolite was increased or decreased in recovered COVID-19 patients when compared with healthy controls, respectively.

We found that the levels of 22 metabolites, such as xanthosine, hypoxanthine, and arachidonic acid (AA), were significantly elevated in the peripheral blood of recovered COVID-19 patients compared to HC (Fig. 3). Whereas the levels of 30 metabolites, such as taurine, serotonin, inosine, and adenosine, were reduced in recovered COVID-19 patients compared to HC (Fig. 3). To further understand these metabolic changes, pathway analysis was performed to know the biological implications of these differential metabolites. Pathway analysis revealed that these metabolic alterations in recovered COVID-19 patients were mainly related to purine pathways, amino acids, lipids, amine metabolism (Fig. 4).

### Correlations between DTI network metrics, the various DTI-derived indices, and the metabonomics characteristics

**Fig. 5:**
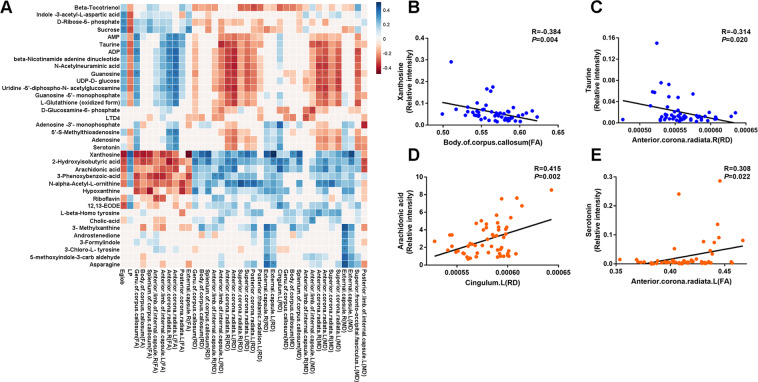
Correlations between DTI network metrics, the various DTI-derived indices, and the metabonomics characteristics. **A** Summary of the correlations between DTI network metrics, the various DTI-derived indices, and the metabonomics characteristics. The color indicates the value of the correlation coefficient of the corresponding correlation (blue, and red are negative and positive correlations, respectively). Correlation with a two-sided *α* of <0.05 was considered statistically significant and marked with an asterisk. **B** Scatter plot of xanthosine and body of corpus callosum (FA). **C** Scatter plot of taurine and right anterior corona radiate (RD). **D** Scatter plot of arachidonic acid and left cingulum (RD). **E** Scatter plot of serotonin and left anterior corona radiate (FA). AMP adenosine 5’-monophosphate, ADP adenosine diphosphate, LTD4 leukotriene D4, 12, 13-EODE 12, 13-epoxyoctadec-97-enoic acid, *R* Pearson’s correlation coefficient.

As displayed in Fig. 5, most differential metabolites displayed significant association with the level of Eglob, Lp, FA, RD, or MD. Many metabolites presented a similar or inversed relationship in FA, RD, or MD. For example, hydroxyisobutyric acid, AA, xanthosine were negatively correlated with FA, inversely positively associated with RD and MD. Among these correlations, the alterations of many metabolites in purine metabolism, such as xanthosine, hypoxanthine, adenosine, inosine, guanosine, and 3-methylxanthine, showed significant correlations with the changes of Eglob, Lp, FA, and RD values in multiple WM tracts, including the corpus callosum, corona radiate, posterior thalamic radiation, internal capsule and external capsule (Fig. 5). We observed lower FA in the left anterior corona radiate of recovered COVID-19 patients showed significant positive correlations with the serotonin level (Fig. 5).

## Discussion

To our knowledge, this is the first study to investigate the mental health alterations in recovered COVID-19 patients without previous underlying diseases. The most prominent finding is the recovered COVID-19 patients showed decreased FA, increased MD, and RD values in widespread brain regions, which showed significant correlations with the PCL-C, PTSD-SS, and GAD-7 values. And graph theory analyses revealed that recovered COVID-19 patients exhibited significantly lower Eglob and longer Lp than HC, which showed significant correlations with the PCL-C. Our results also demonstrate that the plasma metabolic profiling in recovered COVID-19 patients was still obviously different from that in HC even at 3 months after their hospital discharge. Pathway analysis revealed that several pathways involved in purine pathways, amino acids, lipids, amine metabolism were significantly perturbed in the recovered COVID-19 patients. To our knowledge, COVID-19 pneumonia involves multiple systemic immune and inflammatory responses. A previous study also reported neuropsychiatric sequelae of COVID-19 related to their neuroimmune status^18^. So the relation between the mental health alterations, metabolic alterations, systemic immune and inflammatory responses in recovered COVID-19 patients needs to be discussed. Our results have important implications for the pathophysiology of the mental health alterations in recovered COVID-19 patients and novel therapeutic treatment development.

We used a metabolomics approach to investigate the potential molecular mechanisms of the mental health alterations in recovered COVID-19 patients. Our results illustrated that the purine pathways were seriously disturbed in recovered COVID-19 patients when compared with HC, which may be related to the mental health alterations of recovered COVID-19 patients. A previous study showed elevated exhaustion levels and reduced functional diversity of T cells in peripheral blood plays an important role in the development of COVID-19 pneumonia^19^. Broad and strong memory CD4+ T cells induced by SARS-CoV-2 in UK convalescent individuals following COVID-19 were also been reported^20^. We, therefore, speculate the purine pathways of recovered COVID-19 patients may be related to the role of CD4+ T cells. The recovered COVID-19 patients had an increased xanthosine and hypoxanthine, decreased adenosine, inosine, guanosine, and 3-methylxanthine versus HC, which showed significant correlations with the Eglob, Lp, FA, and RD values in multiple WM tracts, including the corpus callosum, corona radiate, posterior thalamic radiation, internal capsule, and external capsule. Peripheral CD4+ T cell-derived xanthine acts on oligodendrocytes in the left amygdala via adenosine receptor A1^21^, and the amygdala plays an acritical role in generating fear and persistent anxiety. Peripheral CD4+ T cells as pivotal mediators of stress-induced mood disorders CD4+ T cells also play an essential role in stress-induced anxiety-like behavior^21^. Thus, purine pathways could provide important information for pathophysiological mechanisms underlying mental health alterations in recovered COVID-19 patients.

Lipids metabolism was also significantly perturbed in the peripheral blood of recovered COVID-19 patients in our study. We found elevated AA significantly correlated with decreased FA, increased MD, and increased RD values in certain brain regions of recovered COVID-19 patients, such as the left cingulum, left anterior corona radiate, and left internal capsule. The cingulum is one of the major fiber tracts for communication within the limbic system, which involved in the regulation of emotions, such as anxiety and PTSD. And the cingulum bundle supports functional interactions that are necessary for fear learning and memory processes^22^. AA is an integral constituent of the biological cell membrane and modulates the function of ion channels and several receptors and enzymes, so AA is necessary for the function of all cells, especially in the nervous system and immune system^23^. Leukotriene B4 (LTB4) is one of the metabolites of AA^24^, physical stress-induced LTB4 triggers severe mitochondrial fission in CD4+ T cells, which further leads to a variety of behavioral abnormalities, such as anxiety^21^. AA level was significantly inversely related to risk for PTSD^25^. A previous study reported no inter-group differences in AA between PTSD patients and HC, which may be attributed to a small sample size and dietary assessment^26^. Thus, Lipids metabolism may provide interpretation for the mental health implications in recovered COVID-19 patients.

We observed lower FA in the left anterior corona radiate of recovered COVID-19 patients, which showed significant positive correlations with the serotonin level. A previous study also found serotonin is decreased in COVID-19 patients when compared to HC^12^. In the CNS, the contributions of serotonin modulate a broad range of targets including, most notably, hypothalamic, limbic, and cortical circuits linked to the control of mood and mood disorders^27^. Serotonergic cell bodies are clustered in midbrain and brainstem raphe nuclei where they project locally, as well as to the spinal cord and a large number of forebrain targets^28^. Dysregulation of serotonin signaling has also been observed in multiple neurobehavioral disorders^29^, including anxiety and depression. In addition, the immune system communicates to the brain via both humoral and neuronal mechanisms, and that CNS serotonin neurons are a direct or indirect target for these actions^30^. In summary, the abnormal serotonin level may provide an indicator of the deteriorated mental health for recovered COVID-19 patients.

We also observed lower FA and higher RD in the corpus callosum, corona radiate, posterior thalamic radiation, internal capsule, and external capsule in recovered COVID-19 patients, which showed significant correlations with the PCL-C, PTSD-SS, GAD-7 scores, and taurine. Recovered COVID-19 patients in this cohort had a decreased taurine versus HC. Taurine prevents oxidative stress and inflammation and hence acts as an endogenous neuroprotector^31^ by positively modulating GABA_A_ and strychnine-sensitive glycine receptors and inhibiting NMDA receptor activation^32^. Importantly, taurine deficiency has been shown to impair mitochondrial complex I activity, with consequent elevation of the NADH/NAD+ ratio and down-regulation of energy metabolism^33^. Furthermore, anxiety was negatively associated with the nucleus accumbens taurine content^34^, which was consistent with our findings, and taurine treatment has been shown to be effective in reducing anxiety-like behaviors^35^.

On the other hand, the decreased FA increased MD and RD values in widespread brain regions of recovered patients may have reflected the different aspects of WM integrity. FA is an aggregate index of WM integrity, which is thought to reflect fiber density, axonal diameter, and myelination in WM^36^. AD and RD have been identified as reflecting axonal and myelin integrity, respectively, that underlie changes in FA. DTI connectivity can be assessed within key functional brain networks, including those critical to the neurocircuitry involved in emotional expression and regulation. Our study exhibited lower Eglob and longer Lp in WM networks in the patients recovered from COVID-19 than healthy control subjects, which showed significant correlations with the PCL-C. The impaired capacity of information transfer maybe because of the disruption of WM connectivity in the patients recovered from COVID-19.

We also observed decreased FA and increased RD values in the corpus callosum, corona radiate, and left posterior thalamic radiation, which showed significant correlations with the GAD-7 and PTSD-SS scores. The left middle frontal gyrus was identified as the terminal aspect of the thalamic radiation, and the left superior frontal gyrus received the most robust projections from the genu of the corpus callosum and the left anterior thalamic radiation. Findings from this study suggested that WM abnormalities related to the frontal-limbic system could be directly related to PTSD and anxiety symptoms. However, whether and how WM alterations in this region may change over the longer term in the patients recovered from COVID-19 remains to be determined by longitudinal studies.

Our study has several limitations. First, this is a single-center prospective study with relatively small sample size. Second, the employment of deterministic tractography, which is insufficient in resolving crossed fibers, may result in the loss of fibers. Third, the causality among the cerebral WM changes, metabonomics characteristics, and the mental health implications were not studied.

## Conclusion

We observed multiple alterations in both WM integrity and plasma metabolomics that may explain the deteriorated mental health of recovered COVID-19 patients even at 3 months after their hospital discharge. These findings may provide potential biomarkers for the mental health evaluation for the recovered COVID-19 patients and potential targets for novel therapeutics.

## Supplementary information

Appendix
