# Supplementary material for: Characteristics of mental health implications and plasma metabolomics in patients recently recovered from COVID-19
Source: Transl Psychiatry. 2021 May 21;11:307. doi: 10.1038/s41398-021-01426-3 (PMC8138845; doi:10.1038/s41398-021-01426-3)
Supplement: Supplementary file 1 — Appendix [file 41398_2021_1426_MOESM1_ESM.docx]

Appendix E1

The inclusion and exclusion criteria of the participants

The inclusion criteria were as follows: (1) patients had been discharged from Wuhan Union Hospital 3 months ago; (2) all participants were negative for SARS-CoV-2 nucleic acid as confirmed by realtime polymerase chain reaction at the time of inclusion; (3) all recovered COVID-19 patients who had positive result in serum test of coronavirus IgG antibodies and negative result in serum test of IgM antibodies of SARS-CoV-2. Participants were excluded if they had any of the following: underlying neurological diseases (such as cerebrovascular lesions), a history of comorbidities (such as diabetes, hypertension and malignancy), individuals with current or past psychiatric illness, and a history of alcohol or drug abuse, and any contraindications to MRI.

Image Acquisition
All of the participants were imaged with a 3-T MR imager (Magnetom Skyra; Siemens, Erlangen, Germany) in the Department of Radiology, Union Hospital, Tongji Medical College, Huazhong University of Science and Technology. Foam pads and headphones were used to minimize head movement and image noise. DTI images were collected axially by using a single-shot echo-planar sequence: repetition time/echo time = 11900 ms/95 ms, flip angle = 90°, field of view = 224 × 224 mm^2^, matrix size = 122 × 122, 60 axial sections, section thickness = 2 mm, voxel size = 1.84 × 1.84 × 2 mm^3^; 64 gradient directions with b value of 1000 sec/mm^2^ and one image with a b value of 0 sec/mm^2^; and three averages. Three-dimensional T1-weighted images were acquired at the sagittal plane by using a magnetization-prepared rapid gradient echo sequence: repetition time/echo time = 2400/2.26, flip angle = 8°; inversion time = 950 ms, field of view = 256 × 256 mm^2^, matrix size = 256 × 256, 176 sagittal section, section thickness = 1 mm, voxel size = 1 × 1 × 1 mm^3^. Additionally, an axial fluid-attenuated inversion recovery and T2-weighted sequence were also collected of each participant for clinical diagnosis. All images were reviewed independently by two experienced neuroradiologists with more than 20 years of experience in clinical radiology. Patients with abnormal MR signals, such as those derived from cerebral infarctions and brain tumors, were excluded from the subsequent analysis.

All images were reviewed independently by two experienced neuroradiologists (CSZ and LY) with more than 20 years of neuro-radiology experiences. Patients with abnormal MR signals, such as those derived from cerebral infarctions and brain tumours, were excluded from the subsequent analysis.

Data Preprocessing

The preprocessing of DTI MR imaging data was performed with FSL software (<http://www.fmrib.ox.ac.uk/fsl/>), as previously described ^1^. Data preprocessing of various DTI-derived indies (FA: fractional anisotropy; MD: mean diffusivity; AD: axial diffusivity and RD: radial diffusivity) was performed using tract-based spatial statistics (TBSS) ^2^ and FSL ^3^. The diffusion-weighted images were corrected for eddy current distortions and head motion with the FDT toolkit available in FSL ^4^. First, the FA images were created by fitting a tensor model to the raw diffusion data using FDT, followed by the brain extraction process using BET ^5^. Second, the FA data from all subjects were aligned to the MNI space. Third, a mean FA image was generated to produce a mean FA skeleton, which represented the centres of all tracts common to the group. For the FA skeleton generation, a FA threshold of 0.2 was used to exclude voxels which are primarily gray-matter or cerebrospinal fluid. Finally, each subject’s aligned FA data were projected onto the skeleton, and the resulting data were fed into voxelwise cross-subject statistical analyses. In the same way, the MD, AD and RD images were aligned to the MNI space and then were projected onto the mean FA skeleton by using the protocol for non-FA images in TBSS. The permutation methods in FSL (the FSL randomise procedure) were used to test FA differences between groups. The WM integrity differences were investigated by using the threshold-free cluster enhancement at P value < 0.05 (5000 permutations) fully corrected for multiple comparisons. A similar tract-based analysis procedure was applied to the MD, AD and RD images. More detailed processing of the various DTI-derived indices can be seen in our previous study ^1^.

Deterministic DTI tractography was perfomed with the Diffusion Toolkit and Trackvis (https://www.nitrc.org/projects/trackvis). Deterministic tractography proceeded until either it turned an angle greater than 45° or the FA was less than 0.2 using the Fiber Assignment by Continuous Tracking algorithm ^6^. We parcellated the individual brain into 90 structures defined in the Automated Anatomical Labeling (AAL) template ^7^, which is widely used in brain network studies because of its computational tractability and reproducibility^8^. In native diffusion space, 2 regions were considered structurally connected if at least 1 fiber bundle with 2 endpoints was located in the 2 regions. We calculated the FA values between the end-nodes as its weight and obtained a weighted symmetrical anatomical 90 × 90 matrix for each subject ^9^.

Network Construction and Analysis

Network construction was performed using the GRETNA Toolbox (<http://www.nitrc.org/projects/gretna/>) as our previous study ^10^. Graph theoretic analyses were performed on the interregional connectivity matrices (weighted undirected networks) to characterize the topological properties of the brain network.

To characterize the global properties of the brain functional networks, following global parameters were used: the clustering coefficient (Cp), the characteristic path length (Lp), the global efficiency (gE), the local efficiency (locE), the normalized clustering coefficient (g), the normalized characteristic path length (l), and the small-worldness s ^11-13^. For more details on interpretation of the above network measures, see reference ^12^.

To assess the statistical significance of between-group comparisons of the network metrics and the measures of various DTI-derived indices (FA, AD, RD and MD), permutation-based non-parametric inferences were performed between the two groups (5,000 random permutations) with the PHQ-9 9, GAD-7 7, HAMA, HAMD, PCL-C scores and age as covariates. For each network metric, we used a nonparametric permutation testing procedure. For each metric, the data labels were randomly reassigned between the 2 groups and t values were computed for each relabeling, for a total of 5000 permutations. P values were calculated on the basis of the distribution of t values obtained from the permutations and were adjusted for multiple comparisons with a false-discovery rate correction. For the multiple comparison corrections, the threshold-free cluster enhancement (TFCE) with FWE (p<0.05) was employed.

Determination of LC/MS

Separation was performed using ACQUITY UPLC HSS T3 column (1.8 μm, 100mm×2.1mm ID) (Waters, USA) (35°C) and the flow rate was 0.35 mL/minute. Positive and negative acquisition modes used the same gradient but different aqueous solution. The mobile phases A and B in the positive ion mode contained 0.1% formic acid in ultrapure water and 100% methanol respectively. The mobile phases A and B in the negative ion mode contained 10 mM ammonium formate and 95% methanol containing 10 mM ammonium formate respectively. Using the following gradient to scan positive and negative ions separately: 10% B (0 minute), 10% B (1 minute), 98% B (13 minutes), 98% B (18 minutes), 10% B (18.5 minutes), 10% B (20 minutes). Using Q Exactive full scan to obtain metabolic data and data-dependent Acquisition method to obtain MS/MS data. The collision energy was 3.8kV and 3.2kV for positive ions and negative ions respectively.

References

1 Li, L. *et al.* Abnormal brain structure as a potential biomarker for venous erectile dysfunction: evidence from multimodal MRI and machine learning. Eur. Radiol. 28, 3789-3800 (2018).

2 Smith, S. M. *et al.* Tract-based spatial statistics: voxelwise analysis of multi-subject diffusion data. Neuroimage**.** 31, 1487-1505 (2006).

3 Smith, S. M. *et al.* Advances in functional and structural MR image analysis and implementation as FSL. Neuroimage**.** 23, S208-S219 (2004).

4 Jenkinson, M., Bannister, P., Brady, M. & Smith, S. Improved optimization for the robust and accurate linear registration and motion correction of brain images. Neuroimage. 17, 825-841 (2002).

5 Smith, S. M. Fast robust automated brain extraction. Human brain mapping. 17, 143-155 (2002).

6 Mori, S. *et al.* Imaging cortical association tracts in the human brain using diffusion-tensor-based axonal tracking. Magn. Reson. Med. 47, 215-223 (2002).

7 Tzourio-Mazoyer, N. *et al.* Automated anatomical labeling of activations in SPM using a macroscopic anatomical parcellation of the MNI MRI single-subject brain. Neuroimage. 15, 273-289 (2002).

8 Chen, H. J., Shi, H. B., Jiang, L. F., Li, L. & Chen, R. Disrupted topological organization of brain structural network associated with prior overt hepatic encephalopathy in cirrhotic patients. European radiology. 28, 85-95 (2018).

9 Zhang, Z. *et al.* Altered functional-structural coupling of large-scale brain networks in idiopathic generalized epilepsy. Brain**.** 134, 2912-2928 (2011).

10 Xu, H. *et al.* Disrupted functional brain connectome in unilateral sudden sensorineural hearing loss. Hear Res. 335, 138-148 (2016).

11 Bullmore, E. & Sporns, O. Complex brain networks: graph theoretical analysis of structural and functional systems. Nat. Rev. Neurosci. 10, 186-198 (2009).

12 Rubinov, M. & Sporns, O. Complex network measures of brain connectivity: uses and interpretations. Neuroimage. 52, 1059-1069 (2010).

13 Bai, F. *et al.* Topologically convergent and divergent structural connectivity patterns between patients with remitted geriatric depression and amnestic mild cognitive impairment. J. Neurosci. 32, 4307-4318 (2012).

**Appendix Table S1**. Summary of the laboratory findings of recovered COVID-19 patients and healthy controls.

| Laboratory findings | Recovered COVID-19 patients (n=28) | Healthy controls (n=27) | *P*-value |
| --- | --- | --- | --- |
| CRP, mg/L | 0.7 (0.32-1.50) | 0.71 (0.3-1.14) | 0.81^2^ |
| WBC, × 10⁹ per L | 5.20 ± 1.10 | 5.73 ± 0.80 | 0.049^1^ |
| RBC, × 10^12^ per L | 4.80 ± 0.48 | 4.85 ± 0.58 | 0.73^1^ |
| Haemoglobin, g/L | 147.96 ± 14.45 | 147.52 ± 15.92 | 0.91^1^ |
| MCV, fl | 91.95 (89.28-93.18) | 92.8(88.3-96.45) | 0.60^2^ |
| MCH, pg | 30.9 (30.4-31.3) | 31.2(29.6-32.65) | 0.490^2^ |
| MCHC, g/L | 337 (334-339) | 338 (333-342.5) | 0.698^2^ |
| Platelets, × 10^9^ per L | 232.64 ± 55.94 | 228.11 ± 50.17 | 0.75^1^ |
| Neutrophils, ×10^9^ per L | 3.02 ± 0.76 | 3.45 ± 0.80 | 0.046^1^ |
| Lymphocytes, ×10^9^ per L | 1.77 ± 0.51 | 1.83 ± 0.55 | 0.64^1^ |
| Monocytes, × 10^9^ per L | 0.28 ± 0.08 | 0.30 ± 0.08 | 0.32^1^ |
| Eosinophils, × 10^9^ per L | 0.08 (0.06-0.12) | 0.09 (0.06-0.15 ) | 0.75^2^ |
| Basophils, × 10^9^ per L | 0.01 (0-0.01 ) | 0.01 (0.01-0.01 ) | 0.092^2^ |
| MPV, fl | 9.45 (9.18-10.18) | 9.5 (9.05-10.25 ) | 0.99^2^ |
| AST, U/L | 22.5 (19-31.25) | 21 (17-23.5) | 0.095^2^ |
| ALT, U/L | 26 (15.75-45.25) | 19 (15-27) | 0.117^2^ |
| AST/ALT | 0.94 ± 0.34 | 1.03 ± 0.38 | 0.324^1^ |
| ALP, U/L | 54 (46.75-71.25) | 63 (54-80.5) | 0.089^2^ |
| LDH, U/L | 183.79 ± 37.93 | 167.37 ± 23.56 | 0.059^1^ |
| γ-GT, U/L | 19.5 (13.75-30.25) | 17 (13-25) | 0.555^2^ |
| TBIL, µmol /L | 12.45 (10.4-14.7) | 11.8 (9.5-14.8) | 0.55^2^ |
| DBIL, µmol/L | 2.95 (2.5-3.65) | 2.7 (2.2-3.9) | 0.474^2^ |
| IBIL, µmol/L | 9.5 (7.38-11.78) | 8.6 (7.5-10.85) | 0.625^2^ |
| Total protein, g/L | 77.31 ± 2.93 | 76.33 ± 3.71 | 0.282^1^ |
| Albumin, g/L | 47.99 ± 2.86 | 46.51 ± 2.34 | 0.042^1^ |
| Globin, g/L | 29.32 ± 2.87 | 29.81 ± 3.52 | 0.568^1^ |
| Albumin/globin | 1.6 (1.5-1.9) | 1.6 (1.45-1.75) | 0.240^2^ |
| BUN, mmol/L | 4.19 (3.88-5.20) | 4.75 (3.99-5.61) | 0.316^2^ |
| Creatinine, μmol/L | 62.6 (55.05-74.78) | 71.1 (63.8-76.6) | 0.056^2^ |
| UA, µmol/L | 365 (295.85-438.78 ) | 320.7 (269.5-385.7) | 0.198^2^ |
| Glucose, mmol/L | 5.09 (4.76-5.45) | 5.22 (4.99-5.4) | 0.337^2^ |
| Mg, mmol/L | 0.87 ± 0.07 | 0.89 ± 0.05 | 0.088^1^ |
| P, mmol/L | 0.97 ± 0.18 | 0.95 ± 0.20 | 0.715^1^ |
| Ca, mmol/L | 2.21 ± 0.06 | 2.19 ± 0.07 | 0.450^1^ |
| Creatine kinase, U/L | 106 (75.25-155) | 97 (76.5-131.5) | 0.717^2^ |
| CK-MB activity, U/L | 11 (9-12) | 13 (9-16) | 0.135^2^ |
| α-HBDH, U/L | 135.5 (123.75-146.5) | 139 (121-147.5) | 0.762^2^ |
| CO_2_, mmol/L | 24.11 ± 2.76 | 24.79 ± 1.72 | 0.278^1^ |
| PT, s | 12.15 (11.6-12.63) | 12.3 (11.75-12.65) | 0.538^2^ |
| INR | 0.92 (0.86-0.96) | 0.93 (0.88-0.97) | 0.538^2^ |
| APTT, s | 36.85 (34.65-40.03) | 37 (35-39) | 0.833^2^ |
| FIB, g/l | 2.86 (2.69-3.10) | 2.82 (2.7-2.99) | 0.705^2^ |
| TT, s | 16.15 (15.8-16.83) | 16 (15.65-16.4) | 0.197^2^ |

Definition of abbreviation: CRP=C-reactive protein, WBC=white blood cell, RBC=red blood cell, MCV=mean corpuscular volume, MCH=mean corpuscular hemoglobin, MCHC=mean corpuscular hemoglobin concentration, MPV=mean platelet volume, AST=aspartate aminotransferase, ALT=alanine aminotransferase, ALP=alkaline phosphatase, LDH=lactate dehydrogenase, γ-GT=γ-glutamyl transpeptidase, TBIL=total bilirubin, DBIL=direct bilirubin, IBIL=indirect bilirubin, BUN=blood urea nitrogen, UA=urine acid, Mg=Magnesium, P=phosphorus, Ca=calcium, α-HBDH=α-hydroxybutyrate dehydrogenase, CK-MB=creatine kinase–myocardial band, PT=prothrombin time, INR=international normalized ratio, APTT= activated partial thromboplastin time, FIB=fibrinogen, TT=thrombin time.

Note: Data are mean±SD, n (%) or median (IQR), unless otherwise specified.

^1^[independent](file:///D:\AppData\Local\youdao\dict\Application\7.5.2.0\resultui\dict\)sample t test; ^2^Mann-Whitney U test.

**Appendix Figure S1.** The flow chart of participants recruitment.
